# Dip-coating electromechanically active polymer actuators with SIBS from midblock-selective solvents to achieve full encapsulation for biomedical applications

**DOI:** 10.1038/s41598-022-26056-7

**Published:** 2022-12-14

**Authors:** Pille Rinne, Inga Põldsalu, Veronika Zadin, Urmas Johanson, Tarmo Tamm, Kaija Põhako-Esko, Andres Punning, Daan van den Ende, Alvo Aabloo

**Affiliations:** 1 Institute of Technology, University of Tartu, Nooruse 1, 50411 Tartu, Estonia; 2 Smart Interfaces & Modules Department, Philips Research, Eindhoven, The Netherlands

**Keywords:** Actuators, Soft materials

## Abstract

Soft and compliant ionic electromechanically active polymer actuators (IEAPs) are a promising class of smart materials for biomedical and soft robotics applications. These materials change their shape in response to external stimuli like the electrical signal. This shape-change results solely from the ion flux inside the composite and hence the material can be miniaturized below the centimeter and millimeter levels—something that still poses a challenge for many other conventional actuation mechanisms in soft robotics (e.g., pneumatic, hydraulic, or tendon-based systems). However, the components used to prepare IEAPs are typically not safe for the biological environment, nor is the environment safe for the actuator. Safety concerns and unreliable operation in foreign liquid environments have been some of the main obstacles for the widespread adoption of IEAPs in many areas, e.g., in biomedical applications. Here we show a novel approach to fully encapsulate IEAP actuators with the biocompatible block copolymer SIBS (poly(styrene-*block*-isobutylene-*block*-styrene)) dissolved in block-selective solvents. Reduction in the bending amplitude due to the added passive layers, a common negative side-effect of encapsulating IEAPs, was not observed in this work. In conclusion, the encapsulated actuator is steered through a tortuous vasculature mock-up filled with a viscous buffer solution mimicking biological fluids.

## Introduction

**Figure 1:**
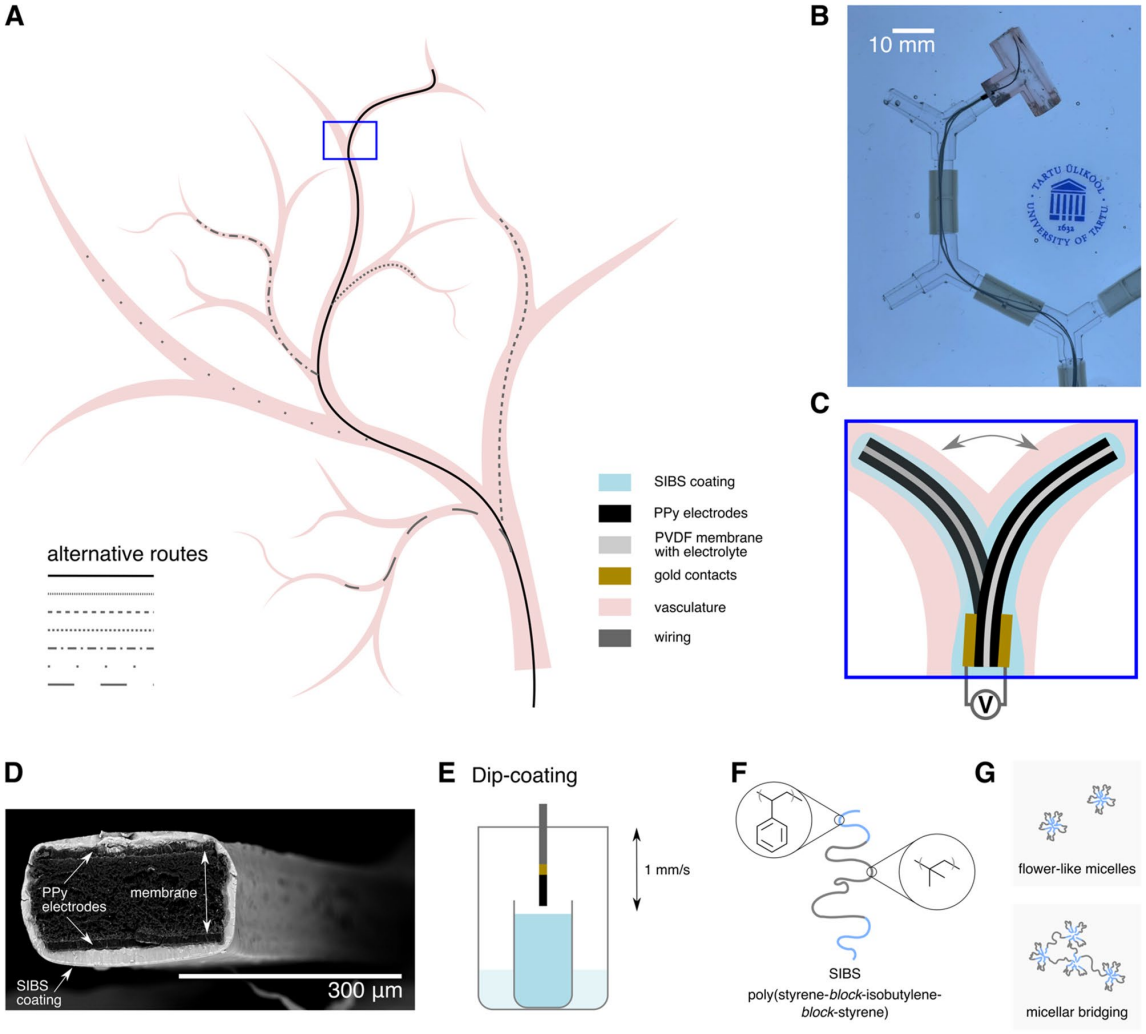
Encapsulated ionic electromechanically active polymer actuator in smart tools for biomedical applications. Steerable actuator navigation in branched channels (**A**). A 20 mm × 1 mm × 150 µm (length × width × thickness) encapsulated polypyrrole (PPy) actuator steered in tortuous vasculature mock-up, snapshot from the Supplementary Video 1 (**B**). Working principle of a trilayer bending type electromechanically active polymer actuator that can be used for navigation in tortuous branching channels as demonstrated in the Supplementary Video 1 (**C**). SEM image of the cross-section of a miniaturized and encapsulated 250 µm × 150 µm (width × thickness) PPy actuator (**D**) showing complete coverage of both the electrode area as well as the actuator's sides. The actuator is dip-coated (**E**) in the solvent atmosphere using 1 mm/s as the dipping and withdrawal speed. Encapsulating polymer (SIBS) structure (**F**) and possible block copolymer chain aggregation behaviours in midblock-selective solvents that could result in improved coating performance without the use of surfactants (**G**).

In recent decades, intravascular interventions have steadily increased in number^1^, also for complex procedures^2,3^. At the same time, patients are becoming older and with more medical comorbidities, resulting in an increase of anatomical complexity and high-risk cases^4^. Often, these difficult cases with narrow, tortuous blood vessels still pose challenges for the skilled professionals when using passive instruments. Both, failure to reach the target as well as delays during the procedure can lead to decreased patient outcomes. Sometimes, robotic catheters^5^ may be used to navigate arduous vasculature, but the use of such instruments is not widespread as safe operation typically requires extensive training to avoid damaging the vessels due to their stiff and non-compliant nature. Smart, compliant and safe tools^6^ that would help to reach the target site faster and with minimal risk of injury would therefore be of high value (Fig. 1A, B).

Ionic electromechanically active polymer actuators (IEAPs) have been regarded as promising materials for smart biomedical applications (e.g. minimally invasive surgical tools like guidewires for steerable catheters) thanks to their low actuation voltage (in the range of a few volts) and soft and compliant nature^7–9^. In the three-layer (electrode-membrane-electrode) configuration, these materials exhibit bending motion caused by the ion flux inside the composite in response to the applied electrical signal (Fig. 1C). Therefore, a controlled electrolytic composition inside these materials is crucial for their reliable operation in biomedical and other devices.

The possibility of using IEAPs in next-generation biomedical devices has driven research towards materials made of fully biocompatible or biofriendly components^10–12^, the possibility of direct operation in more complex electrolytic environments^13–15^, and different encapsulation approaches^16^. While using biocompatible or biofriendly components in IEAPs can be viewed as an added layer of safety, and there is evidence that some IEAPs remain operational also in model biological fluids (e.g., buffers) by using the ions available there instead of an electrolyte tailored to the highest performance, some concerns still remain (e.g., control and performance issues^17^ that are already present in these highly simplified versions of the actual clinical situation in blood). Moreover, safe current levels for devices allowed inside living organisms should also be considered. For example, if a device is placed near the organ of interest (i.e., the microshock situation), then the safe current level is typically cited to be as low as 10 µA^18^. It therefore stands to reason that encapsulation is crucial even for biofriendly IEAPs, as it can provide both ionic as well as electrical insulation of the device.

Preventing drying^19,20^, enabling operation in foreign (liquid) environments^16,21,22^, and more recently also (biological) safety concerns^23,24^ have been relevant in driving the research into barrier coatings for IEAP actuators. The most common encapsulation techniques from simple packaging^20,25,26^ and manual coating^21,22^ using a large variety of materials (e.g., polydimethylsiloxane (PDMS) or polyethylene film) to the more advanced chemical vapor deposition (CVD) of Parylene^19,20,24^ have all been previously tested on different types of IEAP actuators. However, all those approaches have had some drawbacks. The main issues with the method of encapsulation of IEAP actuators so far have involved incomplete layers (e.g., defects, substrate wetting problems resulting in partial coverage). Additionally, a large negative impact on the actuator's deflection due to the added passive (and often too stiff) material/layer(s) has typically previously been observed.

Industrially established dip-coating, spray coating and CVD are the most suitable methods for miniaturized actuators (Fig. 1D) or complex actuator shapes (Supplementary Fig. 1) compared to the more manual ones^16^. Dip-coating (Fig. 1E) is a waste-free industrially established process that offers good control over the resultant film thickness and can be used with a variety of source materials^27,28^, unlike CVD that is compatible only with specific precursors. Dip-coating is also more compatible with objects of complex shape than spray coating, thanks to the possibility to cover areas that are not directly in the line of sight.

Processes related to coating, adhesion and wetting are all ruled by surface effects^29^. Improved wetting in coating applications is often achieved with the help of surfactants^30^. Surfactants are typically amphiphilic compounds that lower the surface (or interfacial) tension between two immiscible liquids (or between liquid and gas or liquid and a solid) and can thus help with wetting or dispersing. It has been previously shown that block copolymers dissolved in solvents selective to only some of the blocks show behaviors similar to those of surfactants^31^. Using a block copolymer dissolved in a suitable selective solvent could improve the encapsulation performance on IEAP devices significantly without the need for additional components like surfactants. As the performance of IEAP actuators can be quite sensitive to possible contaminants, it is favorable when better encapsulation can be obtained without the need to use any extra additives (e.g., surfactants).

SIBS (poly(styrene-*block*-isobutylene-*block*-styrene)) is an ABA-type three-block copolymer with a rubbery midblock (polyisobutylene) and glassy endblocks (polystyrene) covalently bonded to each other (Fig. 1F) resulting in a new material with unique mechanical properties^32,33^. The barrier properties of SIBS are similar to those of butyl rubber (a copolymer of isobutylene and isoprene), but the lack of cleavable moieties in the polymer structure make it more inert^34^. Among styrenic block copolymers, SIBS has the processability, biocompatibility and stability that meet the demanding requirements set for biomedical applications^32^. Moreover, it has already been approved for use in implantable devices^34^. Under mechanical stress, SIBS has the properties of high-strength vulcanized rubber, whereas at elevated temperatures it can be processed like other thermoplastics^35^. Tuning the polystyrene content (commercially typically between 10 to 50%) and the molecular weight (commercially typically between 60,000 to 150,000 g/mol)^34,36^ allows to tune the final properties of the polymer (e.g., mechanical properties^35^ or resistance to degradation^37^).

SIBS dissolved in toluene (a good solvent for both blocks) has been previously used to spray coat polypyrrole (PPy) actuators and resulted in significant improvement of the actuator’s lifetime^23^. However, it was also noted that additional manual intervention to cover the actuators' sides and tip gave the best results, indicating possible partial coverage of the material (possibly due to wetting problems or the inability to spray coat all sides of the actuator equally)^23^. While both blocks of SIBS are soluble in aromatic hydrocarbons (e.g., toluene), only the midblock is also soluble in saturated ones (e.g., alkanes) that are typically considered non-solvents for the styrenic endblocks^38^. It has also been shown that short-chain alkanes (e.g., *n*-hexane and *n*-heptane) exhibit less repulsive interactions with polystyrene^39^ or with the polystyrene block in copolymers^40^ than higher *n*-alkanes. Compared to the solubility of polyisobutylene (the midblock of SIBS) in alkanes, these attractive interactive forces between polystyrene (the endblock of SIBS) and short-chain alkanes are considered less significant, and therefore in this work we consider alkanes to be midblock-selective solvents for SIBS.

Using a selective solvent for one of the SIBS blocks instead of a good solvent for all blocks could cause aggregation (Fig. 1G) of the polymer chain^41–43^ resulting also in a lowered apparent coefficient of surface tension and a better wetting of the substrate during dip-coating. Moreover, it has been shown that in case of block copolymers dissolved in a selective solvent, the non-soluble block preferentially adsorbs and completely collapses on the substrate to form a melt-like thin film layer that could further help to improve the substrate coverage^44^. This behavior seems different from the preferential adsorption of one of the polymer blocks in case of a good solvent, where even the preferentially adsorbed block would still remain swollen while being adsorbed.

In this work we show total encapsulation of bending-type PPy actuators with the biocompatible SIBS dip-coated from a midblock-selective solvent resulting in actuators that are sealed from the environment while showing minimal to no negative influence on the bending performance. The factors affecting the coating drying are studied using the finite element method (FEM) and compared to experimental results using different coating formulations and application speeds. The work continues with the coated actuator performance characterization and coating permeability evaluation. In conclusion, the Supplementary Video 1 shows the actuator steered in tortuous vasculature mock-up that is typically seen as difficult to navigate with common passive tools that would rely on twisting a pre-bent wire for navigation. Because each bend in the vasculature would increase the friction between the passive pre-bent wire and the walls, it is typically increasingly harder to twist (torque) the passive tool with the right precision to prevent injuries as the tool advances. In case of our soft and compliant active steerable material, the bending is controlled solely by voltage.

## Results

The application of coatings is ruled by surface effects. IEAP actuators are complicated substrates to cover given their liquid electrolyte content and surfaces with different properties within the same sample (i.e., from rough and porous on the sides where the membrane is exposed to dense and smooth on the electrode area). The application of a good coating starts with the proper wetting of the substrate that can be achieved by using appropriate solvents or with the help of additives, which we tried to avoid in the current study. Achieving a uniform layer during dip-coating and the layer thickness can be further tuned by adjusting the process parameters (e.g., dipping speed and polymer concentration). Moreover, the safety of the active material during the coating process was investigated/considered.

### Design of an optimal IEAP dip-coating process

The solvent selection for the dip-coating process was evaluated both numerically and experimentally, the polymer concentration, dipping speed and number of applied layers were evaluated experimentally. The pH indicator strips were used as a preliminary model system in our experiments. Similarly to IEAP actuators, pH indicators are made of a porous material and can be cut and shaped as needed. As an added benefit, the coating integrity and possible defects can quickly be evaluated with a dip into acidic or basic solutions.

FEM simulations were performed to theoretically investigate the coating drying behavior during encapsulation of an actuator strip from solvents with different coefficients of surface tension (e.g., toluene or 2,2,4-trimethylpentane, see also Table 1 for the properties of relevant SIBS solvents). The main aim of the numerical analysis was to determine if a full coverage of the actuator could be achieved with available suitable solvents for SIBS or are additional stabilizing effects needed. The simulated cross-sections were compared with cross-sections obtained from SEM imaging of SIBS-coated pH indicator strips.

**Table 1:** Surface tension coefficient values^29^ for possible SIBS solvents and water.

Solvent	Coefficient of surface tension (mN/m at 20 °C)	Solvent type
Water	72.9	Non-solvent
Toluene	28.4	Good solvent for both blocks
Cyclohexane	25	Solvent for both blocks
*n*-heptane	20	Midblock selective solvent
2,2,4-trimethylpentane (isooctane)	18.9	Midblock selective solvent
*n*-hexane	18.40	Midblock selective solvent

**Figure 2:**
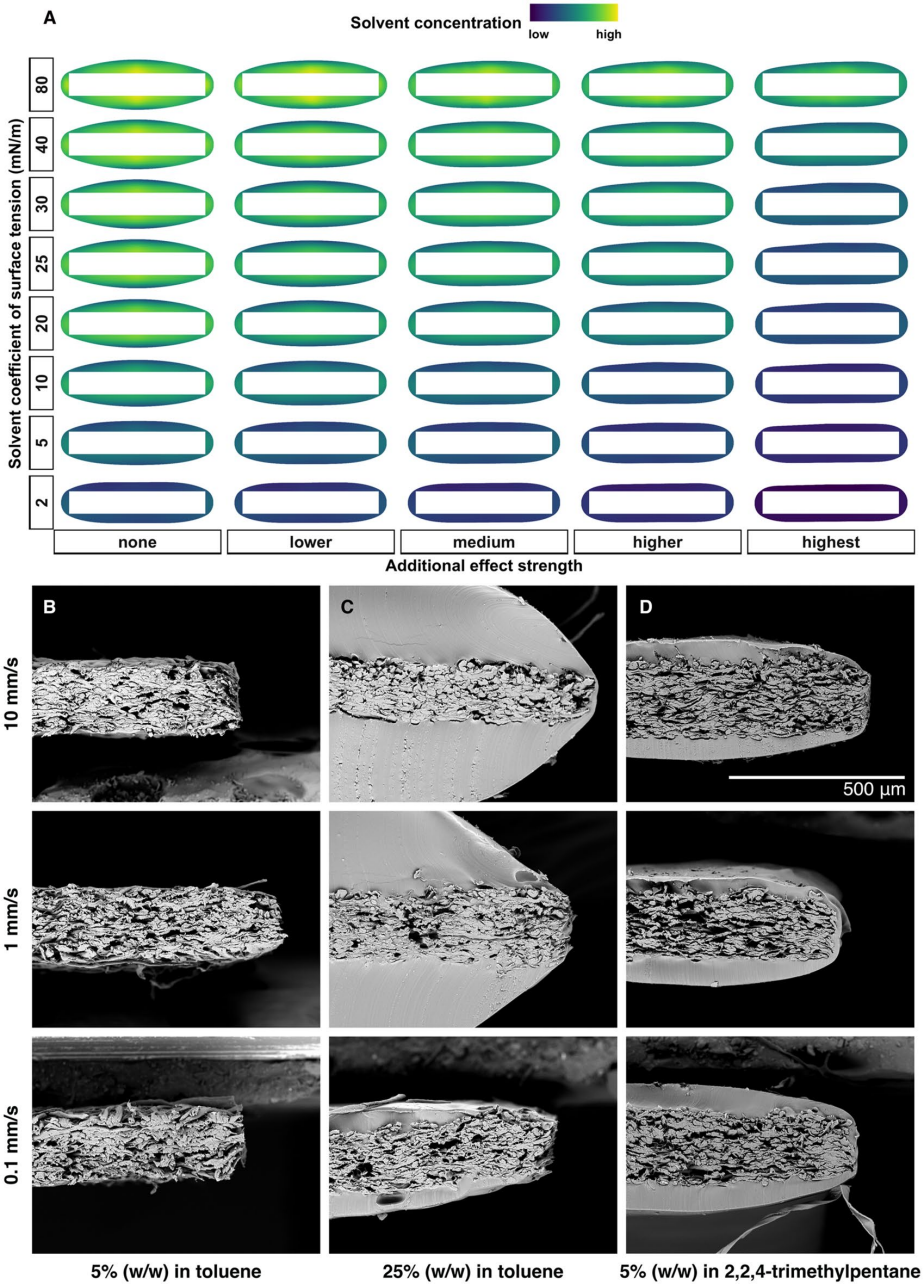
FEM simulation and experimental SEM cross-sections of SIBS coatings from different solvents. FEM heatmaps (**A**) at the time the simulation stopping criterion is reached for scenarios with different solvent coefficient of surface tension values and additional solution stabilizing effect strengths. A high solvent concentration indicates a polymer solution, and a low solvent concentration indicates a solid polymer film. The additional effect strength in FEM simulations is defined as the solvent concentration dependent coefficient of surface tension. In case of the highest additional effect, the coefficient of surface tension is set to approach zero when the solvent concentration approaches zero during drying. In case of no additional effect, the coefficient of surface tension is kept constant during drying. Linear decreases to 0.75, 0.5 and 0.25 of the initial value during drying correspond to the intermediate scenarios of lower, medium, and higher additional effect strength, respectively. Further details on the simulation can be found in the Methods section. SEM cross-sections in **B**, **C**, **D** are obtained by dip-coating pH indicator strips 5 times under the respective solvent atmosphere at different dipping speeds, then cryo-fracturing them in liquid nitrogen and subsequently sputtering with 24 k gold (5 nm) before imaging. Experimental situations showing no coating (**B**) or exposed sides (**C**) of the pH strip could correspond to simulations, where the stopping criterion is reached when the coating formulation still contains a high amount of the solvent. Simulations with the stopping criterion reached later when the coating has almost or fully dried could correspond to an experimental situation where a solvent with extremely low coefficient of surface tension would be used (e.g., 5 mN/m or below) or indicate additional solution stabilizing effects (e.g., when midblock-selective solvents like 2,2,4-trimethylpentane were used (**D**)).

As a general trend, simulations of polymer solutions with solvents having a higher coefficient of surface tension and without any solution-stabilizing effects converged to the simulation stopping criterion early in the drying process, meaning that the liquid layer was broken and the simulation was stopped while the coating was still wet (green and yellow liquid layer, top left area in Fig. 2A). This could indicate an experimental situation where parts of the actuator strip are left uncovered. For example, pH indicator strips coated with 5% (Fig. 2B) and 25% (Fig. 2C) SIBS in toluene resulted in fully uncoated and partially uncoated actuators respectively even after 5 added layers.

On the other hand, simulations of solutions using solvents with lower coefficient of surface tension (even below what is experimentally available) or if additional solution stabilizing effects were introduced, converged later when the coating had already dried (dark blue and purple dry coating layer, bottom right area in Fig. 2A). Finding suitable solvents for SIBS, and available solvents in general, with a coefficient of surface tension significantly below 20 to 25 mN/m might not be possible. Therefore, the simulation results indicated that additional solution-stabilizing effects could be needed to achieve fully covering solid coating layers. A similar trend was observed with pH indicators dipped with 5% SIBS dissolved in the midblock-selective 2,2,4-trimethylpentane where no defects were visible after dipping them into 1 M NaOH after 5 coating layers. Moreover, the SEM cross-sections of the same samples also show a SIBS layer covering all sides of the pH indicator strip (Fig. 2D). The Supplementary Fig. 1 further illustrates that the observed tendencies seem to hold also in case of dip-coating objects of more complex shape (e.g., a spiral).

The dip-coating speed and concentration are two main parameters to control the resulting encapsulant layer thickness. In case of good solvents (e.g., toluene) the experimentally achievable concentrations are higher than in case of selective solvents (e.g., 2,2,4-trimethylpentane). In the latter case, 5% w/w was found to be the maximum concentration where dip-coating soft and compliant materials (e.g., actuators or pH indicator strips) was still experimentally possible. Low concentrations resulted in the need to add multiple coating layers. Figures 2B–D also show the impact of different dipping speeds on the coating thickness after five layers. Since in case of 2,2,4-trimethylpentane, the dipping speed seems to have minor influence on the resulting coating thickness, the subsequent tests with actuators were done using 1 mm/s dipping speed only. The aim was to reduce the submersion time in the coating solution while also reducing the risk of having an uneven coating along the actuator that could be observed during fast dipping speeds.

### Actuator performance and coating barrier properties

**Figure 3:**
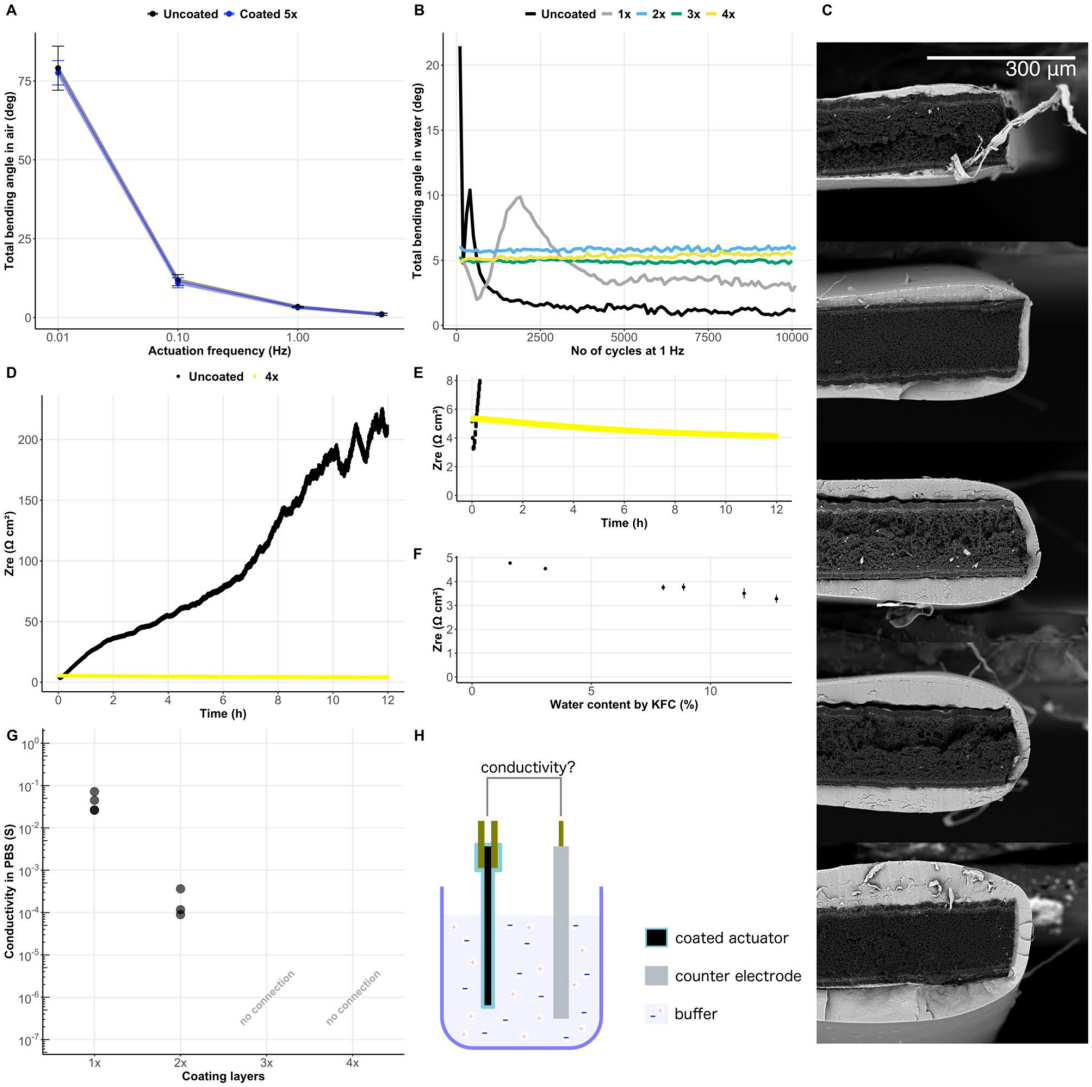
Performance evaluation. Five coating layers show only negligible impact on total maximum deflection of the actuator compared to an uncoated one (**A**). Error bars show one standard deviation of the mean of samples (n_s_ = 4) and cycles (n_c_ = 5). Starting from two coating layers there is no visible change in the maximum amplitude during operation in water over the first 10,000 cycles (**B**). Each dip-coated layer adds to the total coating thickness because the solvent dissolves previously added layers very slowly, as can be seen from SEM images of PPy actuator cross-sections with 1 to 5 added layers (**C** top (1 layer) to bottom (5 layers)). Monitoring electrochemical impedance through the actuator that is submerged in water (500 mL MilliQ) shows electrolyte leach out of the sample in case of uncoated samples and only a small increase in conductivity in case of 4 coating layers (**D**). Small increase in conductivity could be attributed to the reduced viscosity of the electrolyte due to water vapor leaching in the sample (**D–F**). Further studies of the coating permeability while the sample is submerged in a conductive liquid (phosphate-buffered saline) and electrochemical impedance against an external counter electrode (**H**) is measured indicate that three coating layers was sufficient to electrically seal the sample from external electrolytes (**G**), while a small amount of water is still able penetrate the coating due to the characteristic WVTR of SIBS (**D–F**). Sample size in all experiments was 20 (18) mm × 1 mm × 150 µm (L × W × T) with 2 mm mounted between gold contacts and 18 mm free to bend. All samples (excluding uncoated references) were coated with 5% (w/w) SIBS in 2,2,4-trimethylpentane in solvent atmosphere at 1 mm/s dipping and withdrawal speeds.

The dip-coating process and solution formulations presented in this work enable to create fully encapsulated actuators while the passive layers only have negligible negative impact on the actuator’s bending performance. Figure 3A illustrates the bending deformation in response to a triangular actuation signal (± 1 V) at different actuation frequencies before and after five coating layers have been applied. Although it has been previously shown that the actuator's bending performance typically decreases with increasing passive coating layer thickness and that there is a significant trade-off between the maximum achievable deflection and the best barrier properties, this tendency was not observed in the current work. The added passive coating layers did not seem to have a significant negative influence on the deflection of PPy trilayer bending-type actuators. This can probably at least partially be attributed to the low Young's modulus of SIBS (~ 10 MPa^35^, increasing with increasing polystyrene content).

The IEAP encapsulation performance is typically first evaluated via performance in foreign liquid environments. We used the coated actuators and an uncoated reference in MilliQ water (1 Hz, triangular actuation signal ± 1 V) for 10,000 cycles and measured the changes in the bending angle. It appears that at least two coating layers are needed to retain performance over this period (Fig. 3B). In case of a single layer, significant defects were probably present that allowed water to leach in and reduce the viscosity of the electrolyte (corresponding to the initial increase in the bending angle), but then resulted in the electrolyte leach out of the composite and loss of performance (gray line in Fig. 3B) similarly to the uncoated reference sample (black line in Fig. 3B). SEM cross-sections in Fig. 3C further illustrate that the coating thickness increases with added layers. On the one hand this shows that the dip-coating time is short enough and the previously applied layers do not completely dissolve when the next one is added, indicating that the dip-coating process and the used materials are suitable for our actuators. On the other hand, defects that might be present during the first layer could also possibly be covered during the subsequent additions of SIBS.

Although already two coating layers enable actuation for at least 10,000 cycles in a foreign liquid environment, depending on the foreseen application a more sensitive coating impermeability evaluation might be appropriate (e.g., for biomedical devices) to rule out even smaller defects and sources of leaks. Submerging the coated actuator in an ionically conductive solution (e.g., phosphate buffered saline (PBS)) and measuring the ionic conductivity against an external counter electrode (Fig. 3H) is a more sensitive method to test the (im)permeability of a coating. The results in Fig. 3G indicate that at least three coating layers are needed to seal the actuator from the environment. This result further illustrates that with dip-coating SIBS from midblock-selective solvents it is possible to create fully functional encapsulated actuators that seem sealed from the foreign liquid environment even when using more sensitive methods for the evaluation.

Unlike inorganic barrier coatings, polymers are typically more susceptible to water vapor. The ability of water in the form of vapor to escape through a barrier is typically evaluated by standard methods either as permeability or the water vapor transmission rate (WVTR). The WVTR of SIBS is dependent on the polystyrene and polyisobutylene content but has been reported to be 0.24 g*mm/m^2^*day for SIBS with a higher polystyrene content than what was used in this work^45^. Although an exact measurement of permeability or WVTR on the actuator would not be accurate due to the complex geometry and the coating thickness variation, we have still benchmarked the water vapor entering through the coating on the actuator by measuring the resultant change in ionic conductivity inside it over an extended period (Fig. 3D, E). Comparing the changes in ionic conductivity through the actuator to reference samples with a known water content enabled us to estimate the apparent WVTR range through a SIBS coating on an actuator (Fig. 3F and Table S1). The results seem comparable to the reported values of the pristine polymer.

## Discussion

IEAP actuators are typically quite complicated substrates to encapsulate. The surface roughness can be in the same range with the desired coating layer thickness, the surface properties on the electrodes and on the sides vary, and the used electrolytes may be too volatile or a source of contamination. Moreover, high strains are exerted on the capsule during actuation. However, to realize the full potential of IEAP materials in biomedical and other applications, a compatible and industrially scalable encapsulation method is needed. We have shown that dip-coating – an industrially established coating method – is compatible with miniaturized ionic liquid based bending-type conductive polymer actuators. Furthermore, using a midblock-selective solvent in the dip-coating solution, we were able to fully coat the actuators using the biocompatible styrenic block copolymer SIBS.

PPy actuators with SIBS encapsulation dip-coated from a midblock-selective solvent showed extended lifetime in liquid environments with negligible negative impact on the actuation performance from the added passive layers. Moreover, we showed that this capsule is not only extending the actuators’ lifetime in liquid environments by slowing down the electrolyte leach out of the composite but can also be a leak-proof barrier for ionic currents in phosphate buffered saline solutions after enough coating layers have been added. This result enables the use of electrolytes tailored for high-performance actuation in biomedical applications, because these can be sealed off from the organism.

Extended lifetime during operation in foreign liquid environments or in air for water-based actuators has previously been the main performance indicator for IEAP encapsulation. Rarely, SEM images of cross-sections have been used to evaluate the wetting and drying behavior of the coating solution or the presence of any possible defects or uncoated areas. However, it has also been shown previously that actuators with leaking capsules or even no coating at all remain operational in the testing environment (e.g., water) for several hours and/or cycles. In this work we have shown that already a partially impermeable coating is sufficient to provide extended lifetime in foreign liquid environments. However, we have also demonstrated the use of more sensitive methods to evaluate the IEAP coating (im)permeability (e.g., by measuring ionic conductivity or the lack thereof though the capsule) for application areas with higher safety standards.

The results of this study have been summarized in a steerable tool experiment (Supplementary Video 1), where the actuator is successfully navigating through a sequence of branched channels filled with viscous PBS buffer solution to simulate the properties of biological fluids (e.g., blood). Visual feedback through transparent tubing was used during actuation in this model system. In the actual clinical setting, alternative methods (e.g., radio-opaque markers) could be used to determine the location of the actuator tip in the vasculature. Although the added passive encapsulation layers did not have a negative impact on the total bending angle, the addition of further load for visualization purposes could reduce the maximum bending amplitude of the active smart composite material. Therefore, having a coating with minimal to no negative impact on the bending performance is valuable given that in real-world applications there could be a need to add even further functional components.

## Methods

### FEM simulation

The theoretical behavior of different dip-coating solutions during drying was evaluated using the finite element method (FEM) in COMSOL version 5.3a microfluidics module. A two-dimensional approximation of the three-dimensional system was used, i.e., the drying behavior of the liquid layer around the actuator cross-section was investigated assuming symmetry along the actuator. In the beginning of the simulation, a liquid layer composed of solvent molecules uniformly diffused inside the polymer matrix was placed around the actuator cross-section (1 mm × 0.15 mm, width × thickness). The two-phase flow moving mesh interface^46^ was used to model and track the exact position of the interface between the encapsulating formulation and the outer atmosphere during drying. The boundary conditions in case of a moving mesh interface account for surface tension and wetting as well as mass transport across the interface. More information can be found in the Microfluidics Module User's Guide^46^.

Droplet formation, i.e., breaking the liquid layer (or mesh) around the actuator was chosen as the simulation stopping criterion. The final coating was assumed to be a solid polymeric material, unless the simulation stopping criterion was reached before the coating had completely dried. If the simulation stopping criterion was reached when the solvent concentration around the actuator was still very high, then this was interpreted as high probability of observing partial coating or defects (e.g., uncoated sides) experimentally. Whereas, if the simulation stopping criterion was reached when most of the solvent had already evaporated, then this was interpreted as a high probability of observing an intact coating experimentally.

Experimental investigation of the material has shown that there are no changes in the composition of the polymer matrix during dip-coating and drying (i.e., no chemical reactions take place). However, from the experiment we also know that there is a significant change from the initial liquid coating solution layer thickness to the final solid coating due to low experimental polymer concentrations. Considering our simulation approach, significant influence can be expected from this behavior to the diffusion coefficient and viscosity of the coating layer. Thus, the drying effect was simulated by varying both, the diffusion coefficient and viscosity as linear functions between the estimated liquid coating properties and the final solid coating properties.

In this study, the additional coating stabilizing effects were introduced using a concentration-dependent surface tension coefficient. The implication of the surface tension gradient is that it drives motion, known as the Marangoni effect. The surface tension at the interface between the coating and the external atmosphere was simulated as a function of the concentration of the solvent in the polymer matrix. In case of the strongest possible additional effect, the coefficient of surface tension was considered to approach zero as the solvent concentration inside the polymer matrix was zero (i.e., the coating had fully dried). In case of no additional effects, the coefficient of surface tension was kept constant. Linear decreases of the surface tension value to 0.25, 0.5 and 0.75 times the initial value during drying were also evaluated in different simulations.

### Actuator preparation

The polypyrrole (PPy) bending-type actuators were synthesized similarly to Temmer et al*.*^47^ In short, both sides of the commercial polyvinylidene fluoride (PVDF) membrane (Millipore Immobilon-P, thickness ~ 110 µm, pore size 45 µm) were sputtered with 24 k gold (20 nm) using a sputter coater (Leica EM ACE600) to render the membrane electronically conductive for the subsequent electrochemical synthesis. PPy electrodes were electrochemically polymerized on the gold-coated membrane in the cryostat (Lauda Proline) at ‑20 ℃ using Parstat 2273 potentiostat/galvanostat/FRA in galvanostatic mode. The polymerization solution consisted of previously distilled pyrrole monomer (0.2 M) and lithium bis(trifluoromethanesulfonyl)imide (Solvionic, 99.9% extra dry) as the dopant (0.2 M) in propylene carbonate (60 ml). Electrochemical synthesis at lowered temperatures enables to control the PPy deposition rate and achieve higher batch-to-batch reproducibility. Total duration of the electrochemical synthesis was 40,000 s at the current density of 0.1 mA/cm^2^.

After polymerization, the sample was soaked (12 h) and thoroughly washed with MilliQ water and ethanol and then dried *in vacuo* (2 mbar, 24 h). The dry actuators were then soaked in the working electrolyte (ionic liquid 1-ethyl-3-methylimidazolium trifluoromethanesulfonate (EMIM OTf, 99.5%, Solvionic)) for at least 48 h before use. Excess ionic liquid was wiped from the surface of the electrode with filter paper before use. The material was then cut into shape (1 mm × 20 mm) and mounted between custom gold contacts so that 18 mm of the actuator strip was free to bend, and 2 mm was fixed between contacts for electrical connection. The actuators were encapsulated together with contacts. Leaving a part of the actuator uncoated for establishing electrical connection later would lead to leakages near the contacts and prevent accurate performance evaluation because the uncoated part of the actuator near the contacts could be able to bend more than the area under encapsulation. Integrated contacts are also necessary for the safe operation in real-world applications.

### Dip-coating

The encapsulation formulation consisted of SIBS (poly(styrene-*block*-isobutylene-*block*-styrene), SIBSTAR 102 T (pellets) from Kaneka with 15% polystyrene content (w/w%), Mw^37^ 117,000 g/mol) in 2,2,4-trimethylpentane (midblock-selective solvent) or toluene (good solvent for both blocks). All solvents were of analytical grade and used without further purification. The polymer was dissolved by stirring at room temperature in a closed and sealed vial using a magnetic stirrer bar for at least 24 h (resulting in a seemingly homogeneous gel-like substance without any traces of pellets) and then used directly in the encapsulation experiments.

5% (w/w) SIBS dissolved in 2,2,4-trimethylpentane or 25% SIBS dissolved in toluene (10 ml in 15 ml glass vials) were typically used for dip-coating. The goal was to use the maximum concentration that would not cause a deformation of the actuator strip (a soft and compliant material) during insertion into the dip-coating solution, while at the same time reducing the number of layers needed for a complete coating. The maximum usable concentration is significantly lower for midblock-selective solvents (2,2,4-trimethylpentane, *n*-hexane, *n*-heptane) than for good solvents (toluene, tetrahydrofuran, cyclohexane) and therefore the concentrations used here for dip-coating differ.

The dipping and withdrawal speed (0.1, 1 and 10 mm/s) was controlled during dip-coating using a Thorlabs stage. The vial containing the dip-coating solution was placed inside a larger closed glass vessel that contained the same solvent to create a solvent atmosphere and prevent the coating solution in the vial from drying between dips (Fig. 1E). Each layer was dried in the solvent atmosphere for at least 30 min before adding the next.

In addition to actuators, also pH indicators were used as dip-coating substrates for a quick visualization of defects. The encapsulation of actuators and pH indicators used the same coating procedure. The coating defects on pH indicator paper were visualized by dipping the encapsulated indicator strip into 1 M NaOH solution for at least 30 s. Areas in the immediate vicinity of a defect (e.g., typically the tip, edges, corners) changed color, whereas SIBS-coated areas remained pristine.

### Coating objects of more complex shape

A pH indicator paper was cut into 1 mm × 30 mm strips and manually twisted into a spiral around a 21G syringe needle. The spirals were then dip-coated 5 times with SIBS from 25% toluene or 5% 2,2,4-trimethylpentane (isooctane) at 1 mm/s dipping and withdrawal speeds in the corresponding solvent atmosphere. The coated and dried samples, and the uncoated reference were dipped in 1 M NaOH aqueous solution for at least 30 s to check for possible defects (Supplementary Fig. 1).

### Actuator performance evaluation

The actuation performance was evaluated using a triangular potential wave (± 1 V) at different actuation frequencies. The maximum bending angle is known to depend on the actuation voltage (within the safe voltage range) and on the actuation frequency. The bending was monitored using a camera and the overall bending angle was calculated as also described elsewhere in more detail^16,48^. See also Supplementary Fig. 2 for a graphical representation for the experiment. The actuator performance was recorded before and after encapsulation to evaluate the impact of a passive coating on the performance. The actuator’s lifetime in foreign liquid environments was evaluated by actuation in MilliQ water at 1 Hz for 10,000 cycles.

### SEM imaging

The sample cross-sections for scanning electron microscopy (SEM) were prepared using cryo-fracturing as described in detail elsewhere^49^. In brief, the samples were frozen in liquid nitrogen and then cut using a frozen scalpel to obtain clean cross-sections. In case of only partially coated PPy actuators (dip-coated from good solvents), some delamination was occasionally observed during cryo-fracturing. No delamination was observed in case of pH indicator strips or fully coated PPy samples during cryo-fracturing or otherwise. The prepared samples were then attached to a sample holder and sputtered with 5 nm of 24k gold using a sputter coater (Leica EM ACE600). The sputtered cross-sections were imaged using a scanning electron microscope (Hitachi TM3000 with a backscattered electron detector) operated at 15 keV acceleration voltage.

### Capsule permeability evaluation via impedance

The impermeability of the capsule was evaluated in two experimental steps. First, the encapsulated actuator was submerged in ionically conductive buffer solution (phosphate buffered saline^50^) and the electrochemical impedance was measured to evaluate the resistance between the actuator (both electrodes connected together as the working electrode) and an external carbon-based counter electrode (see Fig. 3H). In case of a leak-proof capsule, very high impedance spectra were obtained (indicated with "no connection" in Fig. 3G). In case of a leaking capsule, spectra corresponding to the Randles circuit with measurable ionic conductivity were obtained. The AC voltage for impedance measurements was 5 mV_RMS_. Samples that seemed to be fully isolated were further verified with 50 mV_RMS_ and 100 mV_RMS_ (indicated with "no connection" in Fig. 3G).

Second, the ionic conductivity through the actuator was monitored using impedance while the encapsulated actuator or an uncoated reference sample was submerged in comparably large volume of MilliQ water (500 mL at standard ambient conditions) for 12 h. The electrochemical impedance spectra were measured every minute and ionic conductivity inside the actuator was determined from the high-frequency range of the spectra (overall spectra: 200 kHz–1 Hz, 5 mV_RMS_). In case of a capsule that is perfectly impermeable to water (or water vapor) the internal conductivity is expected to remain constant if temperature is kept constant. However, polymeric materials are typically to some extent permeable to water vapor, which is described by the permeability coefficient or the water vapor transmission rate (WVTR).

The conductivity-increase during the 12-h submersion in MilliQ can be connected to the water content leaking into the actuator through the capsule due to the characteristic permeability properties of the polymer. EMIM OTf solutions with varying water content were prepared (2–15%) to evaluate this possibility. Uncoated electrolyte-less trilayer PPy actuators were first weighed to obtain the dry actuator mass, then soaked in the electrolyte solutions of varying water content for 48 h in closed and sealed vessels. After soaking, the water content in the residual ionic liquid was verified using coulometric Karl Fischer titration (Mettler Toledo), and the ionic conductivity through the soaked composites was measured using electrochemical impedance spectroscopy as described above. The soaked actuators were weighed again to determine the mass of the electrolyte and calculate the water content for the WVTR estimations (Supplementary Table 1). The water content in the residual ionic liquid was assumed to be the same as inside the actuator.

### Performance evaluation in vasculature mock-up

A 1 mm × 20 mm PPy sample with 5 layers of encapsulation was driven with step voltage to navigate an in vitro vasculature mock-up consisting of transparent Y-connectors (internal diameter 4 mm) connected to each other with silicone tubing, and a semi-transparent T-connector at the end that was directly connected to the Y-connectors. The maze was submerged in 1:5 PBS solution in glycerol (v/v) to demonstrate the encapsulated actuator's ability to be steered in a viscous and ionically conductive medium, a model system of biological fluids, such a as blood. The actuator was steered using step voltage (± 1.5 V, with two AA batteries connected in parallel).

## Supplementary Information

Supplementary Video 1.

Supplementary Information 1.

## Data Availability

All data generated or analysed during this study are included in this published article and its Supplementary Information files.
