# Supplementary material for: Dip-coating electromechanically active polymer actuators with SIBS from midblock-selective solvents to achieve full encapsulation for biomedical applications
Source: Sci Rep. 2022 Dec 14;12:21589. doi: 10.1038/s41598-022-26056-7 (PMC9751283; doi:10.1038/s41598-022-26056-7)
Supplement: Supplementary file 2 — Supplementary Information 1. [file 41598_2022_26056_MOESM2_ESM.pdf]

Dip-coating electromechanically active polymer actuators with SIBS from midblock-selective solvents to achieve full encapsulation for biomedical applications

Pille Rinne[1,*], Inga Põldsalu[1], Veronika Zadin[1], Urmas Johanson[1], Tarmo Tamm[1], Kaija Põhako-Esko[1], Andres Punning[1], Daan van den Ende[2], Alvo Aabloo[1]

[1]Institute of Technology, University of Tartu, Nooruse 1, 50411 Tartu, Estonia

[2]Smart Interfaces & Modules Department, Philips Research, Eindhoven, The Netherlands

[*] pille.rinne@eesti.ee

Supplementary Information

Coating objects of more complex shape

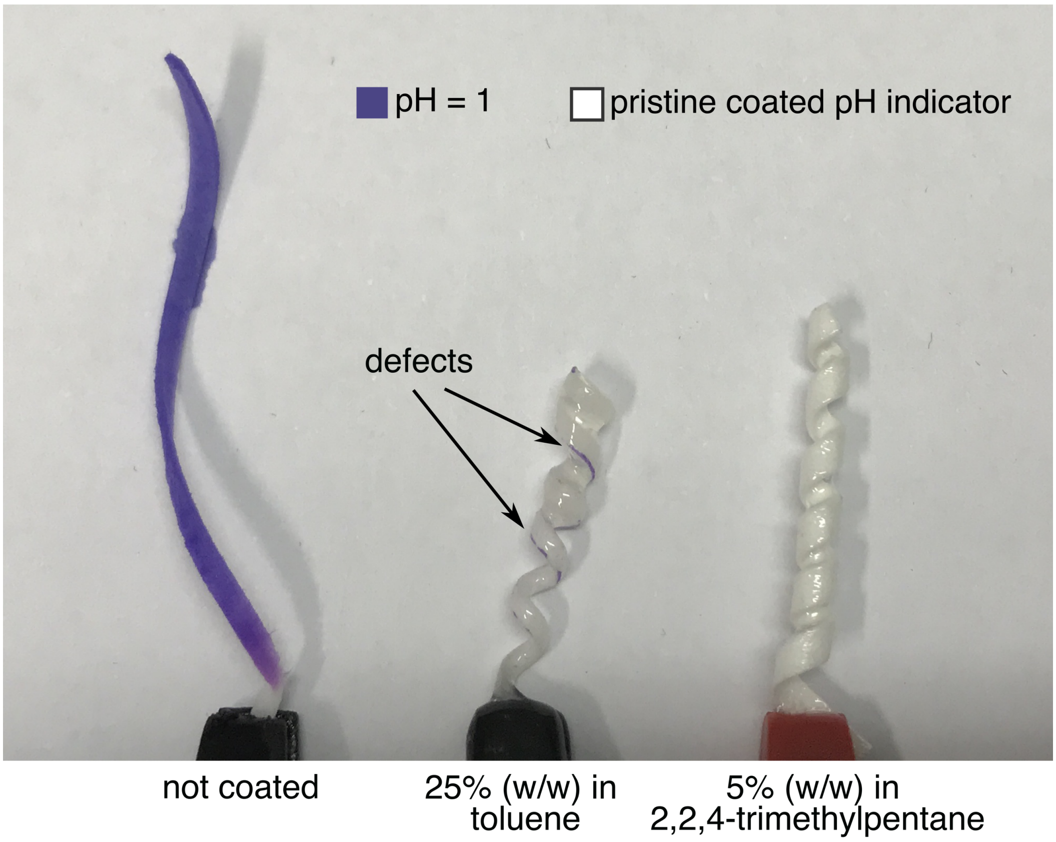

The **Supplementary Figure 1** illustrates that compliant coating can be achieved also on objects with a more complicated shape. The reference sample uncoiled immediately after being dipped into the 1M NaOH solution, whereas the dip-coated samples retained their spiral shape. The sample dip-coated from toluene shows defects around the edges of the pH strip, characteristic for a coating from a non-selective solvent. The sample dip-coated from 2,2,4-trimethylpentane does not show any defects.

Apparent water vapor transmission rate

The coating thickness in case of a 4-times dipped sample is in the range of 10 to 50 μm (see SEM images of actuator cross-sections on **Figure 3C**). On the other hand, **Figure 3E** indicates that after 12 hours of submersion in water, the electrolyte inside the coated actuator could contain between 5-7% of water. Given the sample characteristics in **Table S1**, this indicates 0.11 to 0.16 mg of water. In case of an average coating thickness of 30 μm, this would result in WVTR in the range of 0.16 to 0.22 g*mm/m^2*day that is in comparable to the WVTR reported by Takalloo *et al.*[45] for SIBS with a higher polystyrene content (0.24 g*mm/m^2*day).

Coating area (all sides + tip)	Thickness range from SEM (4 coating layers)	Dry actuator mass (mg), mean (SD)	Actuator with electrolyte (mg), mean (SD)	Electrolyte mass (mg)	Water vapor transmission duration (days)
18 mm x 2.3 mm + 1 mm x 0.15 mm	10 µm - 50 µm	2.94 (0.19)	5.16 (0.15)	2.2	0.5

Table S1. Sample characteristics for the apparent WVTR calculation, n = 5 for the dry and soaked actuator weights showing the mean and one standard deviation of the mean in parenthesis.

Bending angle

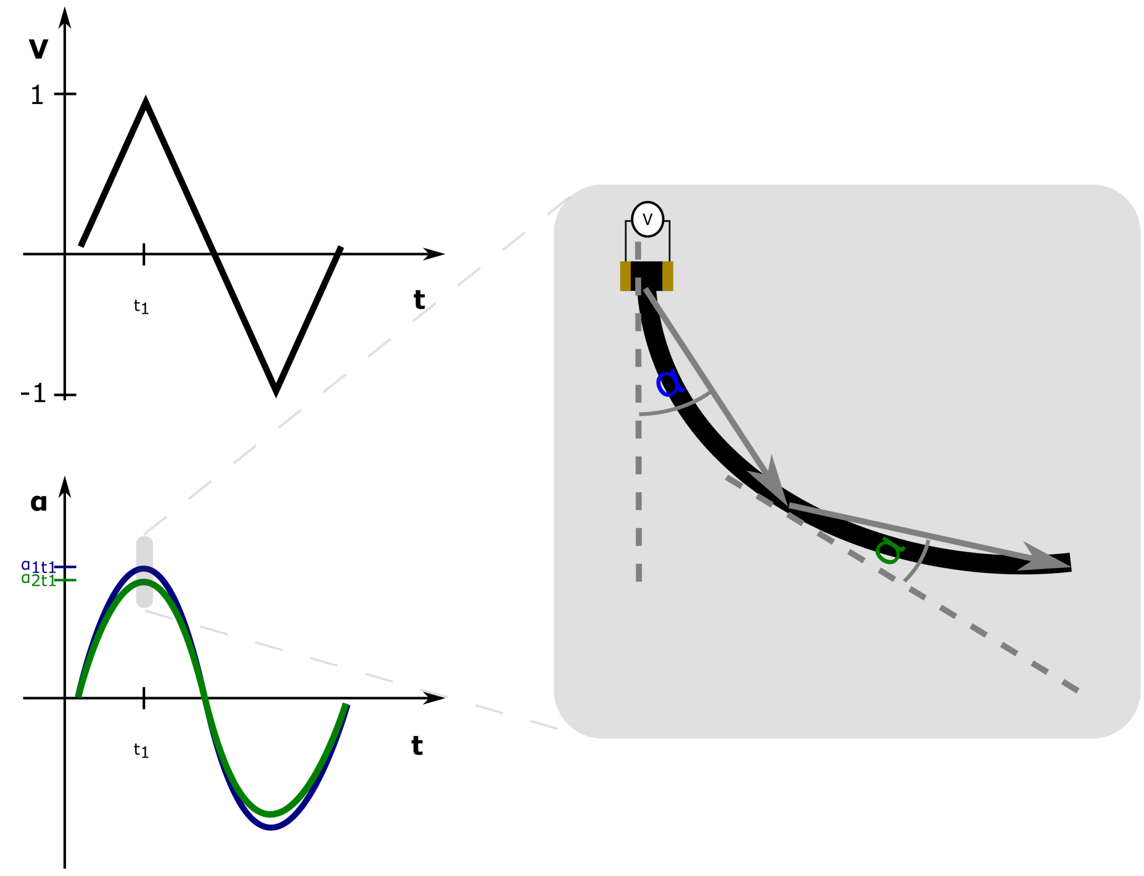

The **Supplementary Figure 2** illustrates the bending angle characterization. The actuator's movement in response to the electrical signal is captured using video. The actuator is divided into multiple segments (2 on the figure inset for simplicity, 6 in the experimental section). The angle between the segment of known length (chord of an arc indicated with the arrow) and the tangent (dotted line) is monitored in time. The inset is from time t_1 where both angles are at their respective maximum value in response to 1 V of input. The sum of all angles (to one side) is used to characterize the actuator.
